# Are Postoperative Infections in the First 12 Months after Wide Resection and Megaprosthetic Replacement Associated with the Survival of Osteosarcoma Patients? Results of a Multicenter Study

**DOI:** 10.3390/cancers14112682

**Published:** 2022-05-28

**Authors:** Christine Schwering, Maya Niethard, Georg Gosheger, Maria Anna Smolle, Frank Traub, Simon Adam, Marcel-Philipp Henrichs, Hans Roland Dürr, Jendrik Hardes, Per-Ulf Tunn, Andreas Leithner, Dimosthenis Andreou

**Affiliations:** 1 Department of General Orthopedics and Tumor Orthopedics, Münster University Hospital, 48147 Münster, Germany; ckebeck@gmx.de (C.S.); georg.gosheger@ukmuenster.de (G.G.); mp.henrichs@googlemail.com (M.-P.H.); jendrik.hardes@uk-essen.de (J.H.); 2 Department of Orthopedic Oncology, Helios Klinikum Berlin-Buch, 13125 Berlin, Germany; maya.niethard@helios-gesundheit.de (M.N.); per-ulf.tunn@helios-gesundheit.de (P.-U.T.); 3 Department of Orthopedics and Trauma, Medical University of Graz, 8010 Graz, Austria; maria.smolle@medunigraz.at (M.A.S.); andreas.leithner@medunigraz.at (A.L.); 4 Department of Orthopedics, Tübingen University Hospital, 72076 Tübingen, Germany; frank.traub@unimedizin-mainz.de; 5 Department of Orthopedics and Traumatology, University Medical Center Mainz, 55131 Mainz, Germany; 6 Department of Orthopedics, Campus Grosshadern, Ludwig-Maximilians University Munich, 81377 Munich, Germany; simon_adam@web.de (S.A.); hans_roland.duerr@med.uni-muenchen.de (H.R.D.); 7 Department of Orthopedic Oncology, University Hospital Essen, 45147 Essen, Germany; 8 Division of Orthopedic Oncology and Sarcoma Surgery, Helios Klinikum Bad Saarow, 15526 Bad Saarow, Germany

**Keywords:** osteosarcoma, postoperative infection, response to chemotherapy, patient survival, immunomodulatory treatments

## Abstract

**Simple Summary:**

Postoperative infection is one of the gravest complications in patients following megaprosthetic replacement due to primary malignant bone tumors. On the other hand, several studies have also suggested that patients with a variety of different cancers may have a better chance of survival following the development of an infection, possibly as a result of the immune and inflammatory host responses to infection. Our retrospective analysis of 437 extremity osteosarcoma patients found that patients with a poor response to neoadjuvant chemotherapy and an infection in the first 12 months after primary tumor surgery had a better chance of survival compared to patients without infections. If this finding can be validated in a different patient cohort, it would suggest that the efficacy of novel immunomodulatory treatments in osteosarcoma patients should be evaluated and reported separately for patients with a good and a poor response to preoperative chemotherapy, as the latter might benefit more from such treatments.

**Abstract:**

Recent retrospective studies suggested that early postoperative infections might be associated with a survival benefit for extremity osteosarcoma patients, but the reported results have been conflicting. The files of 437 patients with a newly diagnosed, high-grade osteosarcoma of the extremities treated at 5 referral centers in Germany and Austria between 1989 and 2016 were retrospectively evaluated. All patients underwent multi-agent chemotherapy and limb-sparing tumor excision, followed by endoprothetic replacement. We used the Kaplan–Meier method to calculate survival curves, which we compared with the log-rank test. With a median follow-up of 100 months (interquartile range, 49–155 months), local recurrence (LR) probability, event-free survival (EFS), and disease-specific survival (DSS) after 5 years in this selected patient cohort amounted to 5%, 67%, and 79%, respectively, and 46 patients (10.5%) developed an early postoperative infection. We found no significant differences in LR, EFS, or DSS between patients with and without early infections, and there were no differences in known prognostic factors between the two groups. However, in subgroup analyses patients with a poor response to neoadjuvant chemotherapy and an early infection had a better DSS compared to patients without early infections (93% vs. 62% after 5 years, *p* = 0.044). Provided that our findings can be validated in separate patient cohorts, we believe that patient outcome after adjuvant immunomodulatory treatments in osteosarcoma patients should be evaluated and reported separately for good and poor responders to neoadjuvant chemotherapy in future studies.

## 1. Introduction

The most common reconstruction method following wide resection of extremity osteosarcomas is megaprosthetic replacement [1,2]. Modern implants offer several advantages, such as a modular design, which allows for the reconstruction of massive bone defects in 1 to 2 cm increments; their wide availability and cost-effectiveness; and immediate stability rendering early weight-bearing possible [1,2,3]. On the other hand, they are also associated with high rates of deep infections, a grave complication that can lead to multiple revision surgeries and, sometimes, a secondary amputation of the affected limb [1,2,3]. However, several studies have also suggested that patients with a variety of different cancers may have a survival benefit following the development of an infection, possibly as a result of the immune and inflammatory host responses to infection [4,5,6,7]. Furthermore, it has been hypothesized that immunomodulatory treatment approaches may lead to improved outcomes in selected subgroups of patients with osteosarcoma. A recent phase II study of the Italian Sarcoma Group demonstrated that the addition of mifamurtide, an agent that increases innate immune response and activates macrophage anti-tumoral phagocytosis, to standard chemotherapy was associated with a superior event-free survival probability in selected high-risk osteosarcoma patients, compared to historic controls [8].

Regarding the impact of postoperative infections in osteosarcoma, following a report by Lascelles et al. demonstrating improved survival in dogs with osteosarcoma that underwent limb-sparing surgery and developed a postoperative infection [9], Jeys et al. published a large retrospective analysis showing that the development of an early periprosthetic infection was associated with improved survival in patients with extremity osteosarcoma [4]. However, a couple of years later an analysis by Lee et al. using a slightly different methodology suggested that the improved prognosis of patients with early infections was likely an expression of more favorable clinical characteristics of the infected patients, rather than a result of the infection itself [10]. Recently, a third, smaller study by Chen et al. reported on excellent overall and event-free survival rates for patients with extremities osteosarcoma developing postoperative infections after limb-sparing surgery, compared to very poor results for patients without infections [11], leading to confusion regarding the possible prognostic effect of infection on osteosarcoma survival. For this reason, we decided to perform a large, multicenter study to evaluate the possible prognostic benefit of an early postoperative infection in osteosarcoma patients undergoing megaprosthetic replacement.

## 2. Materials and Methods

### 2.1. Study Design

We retrospectively reviewed the surgical databases of 5 referral sarcoma centers in Germany and Austria and identified 586 patients with a newly diagnosed, previously untreated, histologically confirmed high-grade osteosarcoma of the extremities, who underwent wide surgical excision of the primary tumor between 1989 and 2016 and achieved complete surgical remission of all macroscopic tumor foci. We only included patients with primary metastases if they achieved complete surgical remission, as survival rates of approximately 50% after 5 years have been demonstrated for this patient group [12]. We excluded 105 patients who did not undergo endoprosthetic replacement, 27 patients who received no pre- or postoperative chemotherapy, and 7 patients with unclear surgical margin status who underwent postoperative radiotherapy. We also excluded 3 patients who died within the first 12 months after surgery and 7 patients with a follow-up of <12 months, leaving 437 patients as the subject of this study. We retrospectively retrieved data regarding patient demographics, the characteristics of the tumor at diagnosis, the treatment of the tumor, the development of postoperative infection and its treatment, and events (local recurrence, metastasis, and disease-related death) that took place during patient follow-up. Al data were anonymized prior to analysis. We conducted the study in accordance with the Declaration of Helsinki, after receiving approval by the local ethics committee.

### 2.2. Definition of Early Periprosthetic Infection

Patients were considered to have a periprosthetic infection if they had clinical (periprosthetic pus, fistula) and paraclinical or culture evidence of infection leading to antibiotic and/or surgical treatment. In order to ensure the comparability of our results to those of previous studies, we defined an early periprosthetic infection as developing within the first 12 months after primary tumor surgery [4,10].

### 2.3. Statistical Analysis

We analyzed contingency tables with the chi-squared test. We used the Shapiro–Wilk test to check continuous variables for normality and calculated medians with their respective interquartile ranges (IQR) for non-normally distributed data. We performed non-parametric analyses using the Mann–Whitney U test. We used the Kaplan–Meier method to calculate survival curves, which we compared with the log-rank test. We performed statistical calculations with the IMB SPSS statistics software version 25.0 (IBM Corp., Armonk, NY, USA). All p values are two-sided; a *p* < 0.05 was considered significant.

## 3. Results

### 3.1. Baseline Demographics

Patients’ demographics, tumor characteristics, and status at last follow-up for all patients, patients with, and patients without an early infection are demonstrated in Table 1. The median patient age at diagnosis was 16 years (IQR, 14–22 years), and the median tumor size amounted to 9 cm (IQR, 7–11 cm). The median follow-up was 75 months (IQR, 37–137 months) for all patients and 100 months (IQR, 49–155 months) for surviving patients. Disease-specific survival (DSS) probability in this selected patient cohort amounted to 95% after 2 years and 79% after 5 years, while event-free survival (EFS) probability was 80% after 2 years and 67% after 5 years. Local recurrence (LR) probability amounted to 3% after 2 years and 5% after 5 years.

A total of 46 patients developed an early postoperative infection after a median time of 2 months (IQR, 1–7 months) after primary tumor surgery. All patients had a deep infection. A pathogen was identified in 40 patients, with coagulase negative Staphylococcus being the most common microorganism, identified in 30 patients, followed by Staphylococcus aureus in 8 patients. These patients underwent targeted antibiotic treatment depending on the results of the antibiotic susceptibility testing, while the remaining patients received empirical antibiotic therapy. The surgical treatment of infection depended on the philosophy of the treating physicians and the clinical findings: 15 patients underwent a one-stage prosthesis exchange, 14 patients underwent a two-stage exchange, while 11 patients underwent radical debridement, antibiotics, and implant retention (DAIR)—no data on the exact type of surgical treatment were available for 6 patients.

Only 8 patients underwent secondary amputation due to infection in our cohort, and only 2 of these amputations took place in the first 12 months after primary tumor surgery—one in a patient with a good response to neoadjuvant chemotherapy, and another in a patient with a poor response.

### 3.2. Prognostic Factors

The influence of known prognostic factors on LR, EFS, and DSS is shown in Table 2. There were no differences in patient age (*p* = 0.365), tumor size (*p* = 0.434), the presence of primary metastases (*p* = 0.326), and the histological response to neoadjuvant chemotherapy (*p* = 0.729) between patients with and without early periprosthetic infections.

**Figure 1:**
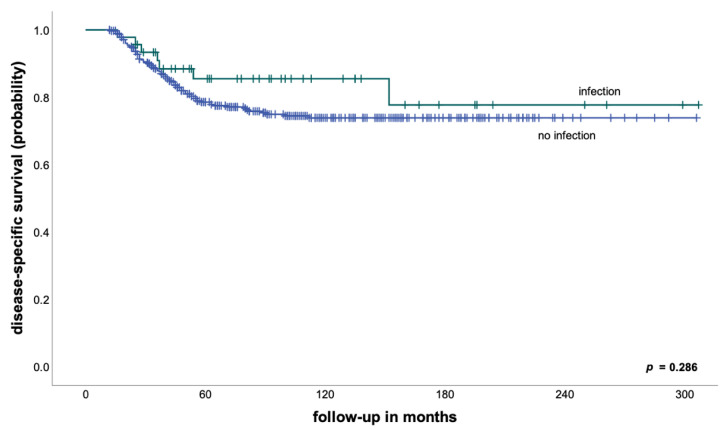
Disease-specific survival in patients with and without early postoperative infections (the entire cohort).

We found no significant differences in EFS (71% vs. 67% after 5 years, *p* = 0.521) and DSS (86% vs. 79% after 5 years, *p* = 0.286—Figure 1) between patients with and without an early periprosthetic infection. However, in keeping with the findings of previous studies [10,11], none of the patients with an early postoperative infection developed a local recurrence in our cohort, compared to an LR of 6% after 5 years for patients without early infections. Although this difference was not statistically significant (*p* = 0.112), taking into consideration the low total number of patients developing a local recurrence in our cohort, multiple studies have shown that the development of local recurrence in osteosarcoma patients is associated both with a poor histological response to neoadjuvant treatment and very poor survival [12,13].

**Figure 2:**
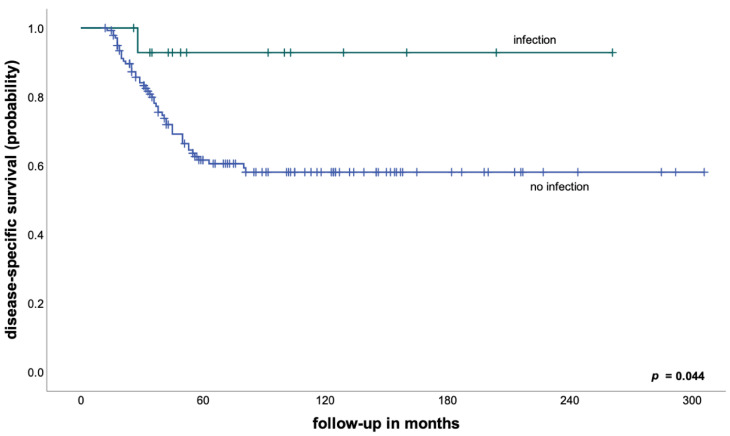
Disease-specific survival in patients with a poor response to neoadjuvant chemotherapy according to the development of an early postoperative infection.

We therefore performed subgroup analyses to examine the prognostic impact of early postoperative infection depending on histological tumor response to neoadjuvant chemotherapy and found that an early periprosthetic infection in patients with a good response was associated with a DSS of 85% after 5 years, compared to 89% for patients without an early infection (*p* = 0.604). On the other hand, patients with a poor response to neoadjuvant treatment and an early infection had a significantly better DSS compared to patients without an early infection (93% vs. 62% after 5 years, *p* = 0.044—Figure 2).

## 4. Discussion

The introduction of adjuvant multiagent chemotherapy in the treatment of osteosarcoma in the 1970s led to dramatic improvements in patient prognosis, with long-term survival rates for localized disease increasing from less than 20% to 65–70% [14]. However, these improvements quickly reached a plateau, and novel approaches are needed for patients at a high-risk for early relapses and disease-related mortality [14,15]. Immunotherapy has long been considered to have a therapeutic potential for osteosarcoma [16], and recent retrospective analyses from surgical databases have examined whether the development of early postoperative infection might be associated with an antitumor activity and an improved patient survival [4,10,11].

In the largest study so far, Jeys et al. evaluated the prognostic impact of early infection in a series of 412 patients with extremity osteosarcoma who underwent megaprosthetic replacement between 1981 and 2001 [4]. The development of infection was associated with improved survival in uni- and multivariate analysis, an effect that the authors attributed to a possible reduction in the incidence of metastases and increased survival from metastases in infected patients [4]. However, this study had several limitations that reduced the validity of the reported results. Data regarding known prognostic factors, such as response to preoperative chemotherapy, tumor stage, and surgical margin width were missing in a large number of cases, while the authors included in their cohort patients with low-grade tumors, as well as patients with positive surgical margins and patients who underwent adjuvant radiation treatment. Indeed, their analysis identified the lack of adjuvant radiotherapy as the most important independent positive prognostic parameter for overall survival—a curious finding that suggests a possible flaw in the methodology that the authors followed [4].

In order to evaluate whether the results reported by Jeys et al. might be attributed to the different clinical characteristics of patients with and without early periprosthetic infections, Lee et al. chose to compare 31 patients with high-grade extremity osteosarcoma who underwent limb-sparing surgery after neodjuvant chemotherapy and developed an early postoperative infection, with 62 patients matched for tumor size, location, and response to neoadjuvant chemotherapy without early postoperative infections [10]. In this study, the development of infection was not associated with a survival benefit. The authors therefore concluded that the reported positive effects of deep infection on survival might be an expression of the more favorable clinical characteristics of the infected patients, rather than a result of the infection itself [10]. However, the sample size of 93 patients was small, and the authors did not report whether their study was powered to detect the impact of known important prognostic parameters, such as a good histological response to neoadjuvant chemotherapy, in their cohort [10].

The latest, to our knowledge, available study on this topic in the literature examined an also relatively small cohort of 125 extremity osteosarcoma patients, the majority of whom underwent biological reconstruction rather than megaprosthetic replacement [11]. Only 6 of these patients developed an early postoperative infection. The results of the survival analysis in this study, showing that infection was associated with an improved prognosis, were rather unusual as patients with infections had a very high 5-year overall survival probability (OS) and EFS of 100%, while patients without infections had a 5-year OS of 54% and a 5-year EFS of 43% [11], which is lower than what one would expect in patients with localized extremity tumors at presentation [12]. Furthermore, the local recurrence rate in patients without infections amounted to 24%, which is much higher than the results reported in recent decades for patients with localized extremity tumors [12,17,18].

In our study, analyzing the largest cohort to date of patients with high-grade, extremity osteosarcoma who underwent neoadjuvant chemotherapy, wide surgical tumor excision with megaprosthetic replacement, and adjuvant chemotherapy, we were unable to find an association between the development of a postoperative infection in the first 12 months after surgery and DSS or EFS. However, in subgroup analyses that took patient response to neoadjuvant chemotherapy into consideration, we found that the development of an early postoperative infection in patients with a poor response to chemotherapy was associated with a significantly higher DSS. We can only speculate on the possible reasons for this finding. Given the retrospective nature of our analysis, we obviously cannot assume causality, and we strongly believe that our observations should be validated in separate patient cohorts. It has been suggested, though, that infection may influence the survival of osteosarcoma patients through the stimulation of the patients’ immune system [4,11], and several immunotherapeutic approaches have been evaluated in the treatment of osteosarcoma patients in recent years [19,20,21,22]. Mifamurtide (L-MTP-PE), a fully synthetic lipophilic analog of bacterial cell walls that can elicit the activation of macrophages and monocytes and lead to the release of cytokines and other proinflammatory molecules, has received marketing authorization in the European Union for the treatment of osteosarcoma patients by the European Medicines Agency based on the results of a phase III trial demonstrating an improved overall survival probability for the combination of mifamurtide and conventional chemotherapy compared to chemotherapy alone [20,21,23]. However, due to concerns regarding the study design and difficulties in interpreting some of the study results, no marketing approval was granted in the United States by the Food and Drug Administration, and further studies are attempting to provide clarity regarding the role of mifamurtide in the first-line treatment of osteosarcoma patients [8,23,24].

Several studies have also evaluated the effectiveness of interferons, a group of pleiotropic cytokines with immunological properties and other potential antitumor effects, in the treatment of osteosarcoma [19,22,25]. Two Scandinavian studies have reported on long-term outcomes of interferon-α as the sole adjuvant treatment to surgery, demonstrating 10-year survival rates of 43% for all patients and 63% in a small subset of consecutive patients who received a higher total interferon-α dose prospectively [22,26]. Based on these results, the first European and American Osteosarcoma Study Group trial (EURAMOS-1) examined whether the addition of a pegylated formulation of interferon-α-2b as maintenance therapy following standard chemotherapy might improve patient outcome but could not show any improvement in event-free or overall survival in the first preplanned intention-to-treat analysis [18]. Interestingly, taking the results of our study into consideration, patients with a poor response to neoadjuvant chemotherapy were not eligible for adjuvant interferon-treatment in this trial [18].

Even if the results of our analysis can be validated in future studies, we obviously do not suggest that postoperative infection might be beneficial even in a subset of osteosarcoma patients following multi-agent chemotherapy and limb-sparing surgery, given the challenges in successfully treating infective complications in sarcoma patients following megaprosthetic replacement and the associated risks for subsequent complications or even amputation of the affected limb [27,28]. However, we propose that studies evaluating immunotherapeutic approaches in the treatment of osteosarcoma patients examine the outcomes of patients with a good or poor response to neoadjuvant chemotherapy separately, in order to determine whether poor responders might be better candidates for immunotherapy than good responders. The aforementioned clinical studies either did not [20] or could not [18,22,26] perform such analyses, and while the current French prospective randomized trial on the role of mifamurtide will recruit poor responders to neoadjuvant chemotherapy, the somewhat small target sample size of the study and the additional inclusion of patients with metastatic disease at presentation could render performing meaningful post-hoc subgroup analyses challenging.

Our study has several limitations. One of the disadvantages of its retrospective design is the possibility of selection bias, and the fact that patients were treated over a period of 27 years in 5 different institutions may have caused some heterogeneity in our cohort. We tried to reduce the impact of these drawbacks by only including patients treated by what is still considered to be the gold-standard of wide surgical resection with pre- and postoperative chemotherapy at experienced tertiary sarcoma centers during a time where patients’ prognosis has essentially plateaued [19]. Another limitation due to the observational nature of our analysis is the possibility of sparse-data bias because the patient groups we compared were not equal in size and adequate patient numbers may have been lacking for some combinations of risk factors and evaluated outcomes [29]. We have therefore attempted to avoid overstating our findings, and we underline the need to validate them in future studies. Finally, it could be hypothesized that the reason why patients with early infections developed no local recurrences might be a high rate of secondary amputations. This is, however, unlikely given the very low rate of secondary amputations in our cohort, especially in the first 12 months after primary tumor surgery. Furthermore, it has been shown that there are no differences in the DSS of osteosarcoma patients undergoing limb-sparing or ablative surgery [12].

## 5. Conclusions

In conclusion, the development of postoperative infection in the first 12 months after wide surgical excision and megaprosthetic replacement in osteosarcoma patients undergoing pre- and postoperative multi-agent chemotherapy was not associated with a higher disease-specific survival probability in our patient cohort. However, we did observe that patients with a poor response to neoadjuvant chemotherapy and an early postoperative infection had a significantly better chance of survival compared to patients who developed no early postoperative infections. Provided that this finding can be validated in separate patient cohorts, we believe that patient outcome after adjuvant immunomodulatory treatments in osteosarcoma patients should be evaluated and reported separately for good and poor responders to neoadjuvant chemotherapy in future studies.

## Figures and Tables

**Table 1:** Patient demographics, tumor characteristics, primary treatment, and status at last follow-up for all patients, patients with, and patients without an early infection.

Variable	All Patients * n * (%)	Infected Patients * n * (%)	Non-Infected Patients *n* (%)	*p*-Value
Eligible patients	437 (100)	46 (11)	391 (89)	
Sex male female	269 (62) 168 (38)	29 (63) 17 (37)	240 (61) 151 (39)	0.874
Age ≤40 years old >40 years old	386 (88) 51 (12)	38 (83) 8 (17)	348 (89) 43 (11)	0.222
Tumor site femur tibia humerus others	253 (58) 124 (28) 57 (13) 3 (1)	29 (63) 16 (35) 1 (2) 0 (0)	224 (57) 108 (28) 56 (14) 3 (1)	0.200
Primary metastases no yes not available	360 (82) 70 (16) 7 (2)	41 (89) 4 (9) 1 (2)	319 (82) 66 (17) 6 (2)	0.326
Pathological fracture no yes not available	380 (87) 51 (12) 6 (1)	42 (91) 4 (9) 0 (0)	338 (86) 47 (12) 6 (2)	0.632
T stage T1 T2 T3 not available	161 (37) 248 (57) 12 (3) 16 (3)	21 (46) 22 (48) 3 (6) 0 (0)	140 (36) 226 (58) 12 (3) 13 (3)	0.196
Tumor response to neoadjuvant chemotherapy good (<10% vital tumor) poor (≥10% vital tumor) not available	270 (62) 155 (35) 12 (3)	29 (63) 15 (33) 2 (4)	241 (62) 140 (36) 10 (4)	0.745
Status at last follow-up no evidence of disease alive with disease died of disease died of other cause	309 (71) 29 (6) 91 (21) 8 (2)	35 (76) 4 (9) 7 (15)	274 (70) 25 (6) 84 (22) 8 (2)	0.521

**Table 2:** The influence of known prognostic factors on patient outcome.

Variable	* n *	%	5-Year LR		* p *	5-Year EFS		* p *	5-year DSS		* p *
			%	SE		%	SE		%	SE	
Age											
≤40	386	88	4	1.1	0.007	68	2.5	0.144	81	2.2	0.063
>40	51	12	12	4.7		58	7.0		70	6.8	
Primary metastasis											
no	360	84	5	1.2	0.977	72	2.5	<0.0001	83	2.1	0.0001
yes	70	16	6	3.5		38	6.2		58	6.7	
Pathological fracture											
no	380	88	5	1.1	0.035	69	2.5	0.003	83	2.1	0.001
yes	51	12	11	4.7		51	7.4		59	7.6	
T stage											
T1	161	38	2	1.2	0.049	77	3.4	0.002	83	3.2	0.074
T2	248	59	6	1.7		62	3.3		77	2.9	
					0.689			0.847			0.273
T3	12	3	10	9.5		58	14.2		90	9.5	
Histological response											
<10% vital tumor	270	64	2	0.9	<0.0001	79	2.6	<0.0001	88	2.1	<0.0001
≥10% vital tumor	155	36	10	2.6		49	4.2		64	4.2	

LR, local recurrence; EFS, event-free survival probability; DSS, disease-specific survival probability; SE, standard error.

## Data Availability

All relevant data can be found in the manuscript.
